# Identification of a Novel Protein Complex Containing ASIC1a and GABA_A_ Receptors and Their Interregulation

**DOI:** 10.1371/journal.pone.0099735

**Published:** 2014-06-12

**Authors:** Dongbo Zhao, Nannan Ning, Zhen Lei, Hua Sun, Chuanfei Wei, Dawei Chen, Jingxin Li

**Affiliations:** 1 Department of Thoracic Surgery, Shandong Cancer Hospital and Institute, Jinan, China; 2 Department of Physiology, School of Medicine, Shandong University, Jinan, China; 3 Department of Anesthesiology, Qilu Hospital, Shandong University, Jinan, China; Dalhousie University, Canada

## Abstract

Acid-sensing ion channels (ASICs) belong to the family of the epithelial sodium channel/degenerin (ENaC/DEG) and are activated by extracellular protons. They are widely distributed within both the central and peripheral nervous systems. ASICs were modified by the activation of γ-aminobutyric acid receptors (GABA_A_), a ligand-gated chloride channels, in hippocampal neurons. In contrast, the activity of GABA_A_ receptors were also modulated by extracellular pH. However so far, the mechanisms underlying this intermodulation remain obscure. We hypothesized that these two receptors-GABA_A_ receptors and ASICs channels might form a novel protein complex and functionally interact with each other. In the study reported here, we found that ASICs were modified by the activation of GABA_A_ receptors either in HEK293 cells following transient co-transfection of GABA_A_ and ASIC1a or in primary cultured dorsal root ganglia (DRG) neurons. Conversely, activation of ASIC1a also modifies the GABA_A_ receptor-channel kinetics. Immunoassays showed that both GABA_A_ and ASIC1a proteins were co-immunoprecipitated mutually either in HEK293 cells co-transfected with GABA_A_ and ASIC1a or in primary cultured DRG neurons. Our results indicate that putative GABA_A_ and ASIC1a channels functionally interact with each other, possibly via an inter-molecular association by forming a novel protein complex.

## Introduction

Acid-sensing ion channels (ASICs) belong to the family of the epithelial sodium channel/degenerin (ENaC/DEG) and are activated by extracellular protons [1]. They are widely distributed within both the central and peripheral nervous systems [2]. The activation of ASICs by protons induces sodium and/or calcium influx, giving rise to depolarization and evoking action potentials in neurons [3].Acid-sensing ion channels(ASICs) are associated with various physiological and pathophysiological functions including regulation of synaptic plasticity [4], perception of pain [5], ischemic death of neurons [6] and the termination of seizures [7]. ASICs were modified by the activation of γ-aminobutyric acid receptors (GABA_A_), a ligand-gated chloride channels, in hippocampal neurons [8]. In contrast, the activity of GABA_A_ receptors were also modulated by extracellular pH [9]–[11]. However, the mechanisms underlying this intermodulation remain .unclear. Megan et. al. identified the α β subunit TM2 residue mediating proton modulation of GABA_A_ receptors [12], [13]. Huang et. al. reported that external protons regulated GABA_A_ receptor function by direct or allosteric interaction with the GABA binding site [14]. But whether there was a direct binding site for proton within the GABA_A_ receptor was so far unknown. We hypothesized that GABA_A_ receptors and ASICs channels might form a novel protein complex and functionally interact with each other. In the study reported here, we found that ASICs were modified by the activation of GABA_A_ receptors either in HEK293 cells following transient co-transfection of GABA_A_ and ASIC1a or in primary cultured dorsal root ganglia (DRG) neurons. Conversely, activation of ASIC1a also modifies the current kinetics of GABA_A_ current. Immunoassays showed that both GABA_A_ and ASIC1a proteins were co-immunoprecipitated mutually either in HEK293 cells following transient co-transfection of GABA_A_ and ASIC1a or in primary cultured DRG neurons. Our results indicate that putative GABA_A_ and ASIC1a channels functionally interact with each other, possibly via an inter-molecular association by forming a novel protein complex. ASIC1a is specifically located in DRG neurons and function as a pain sensor, thus the interaction of GABA_A_ and ASIC1a may contribute to pain sensation.

## Results

### Activation of GABA_A_ receptors inhibits ASIC1a currents in HEK293 cells

**Figure 1:**
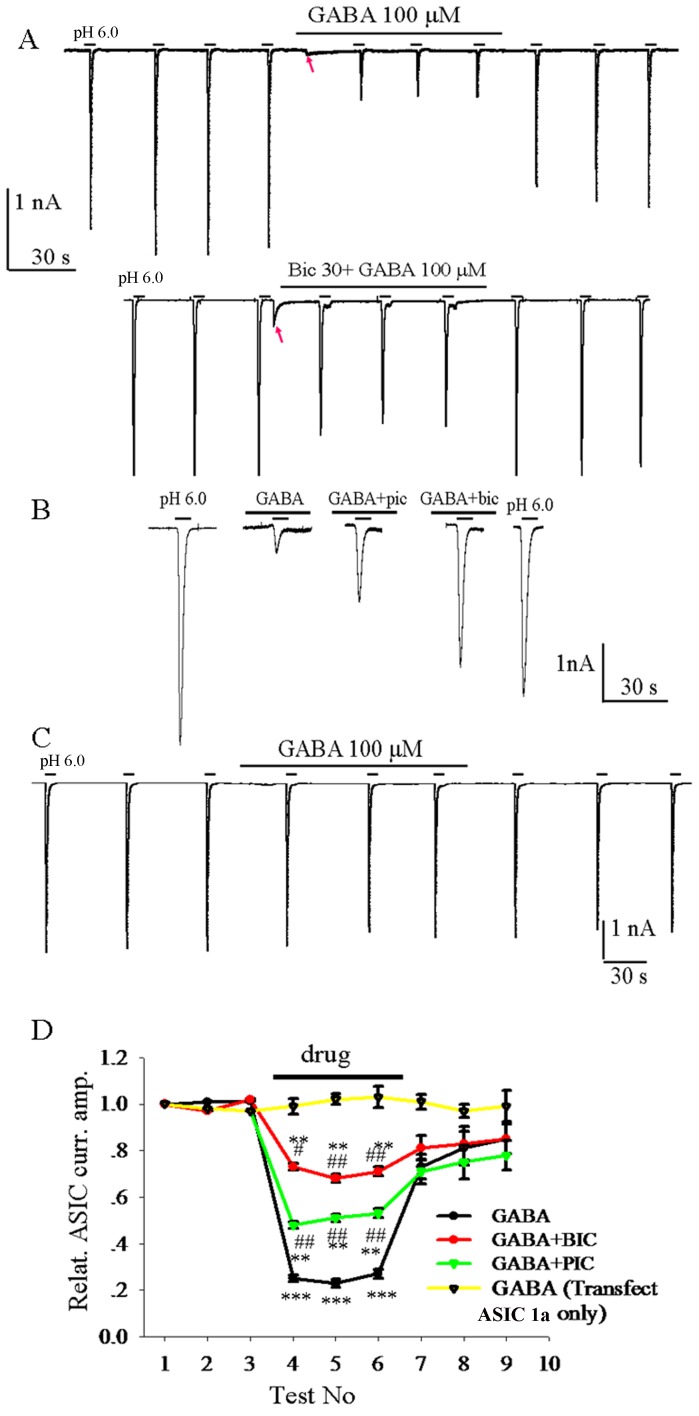
Activation of GABA_A_ receptors reversibly inhibits ASIC1a currents. A, ASIC1a were activated by pH 6.0 solution repetitively in HEK293 cells co-transfected with GABA_A_ receptor subunits (α_1_ and β_2_) and ASIC1a. GABA (100 μM) reversibly attenuated ASIC1a currents. Red arrow indicates the current activated by GABA. B, co-application of bicuculline (BIC, 30 μM) or of picrotoxin (PIC, 100 μM) with GABA largerly abolished the GABA-induced inhibition of ASICs. C, GABA had no effect on ASIC1a currents in HEK293 cells transfected with cDNA of ASIC1a only. D, statistic graph shows relative ASIC currents that were affected by GABA but reversed by antagonists of GABA_A_ receptors. n = 6, ***, *p*<0.001, *T*-test, before vs. after drug; ###, *p*<0.001, *one-way ANOVA*, GABA plus GABA antagonists vs. GABA alone.

**Figure 2:**
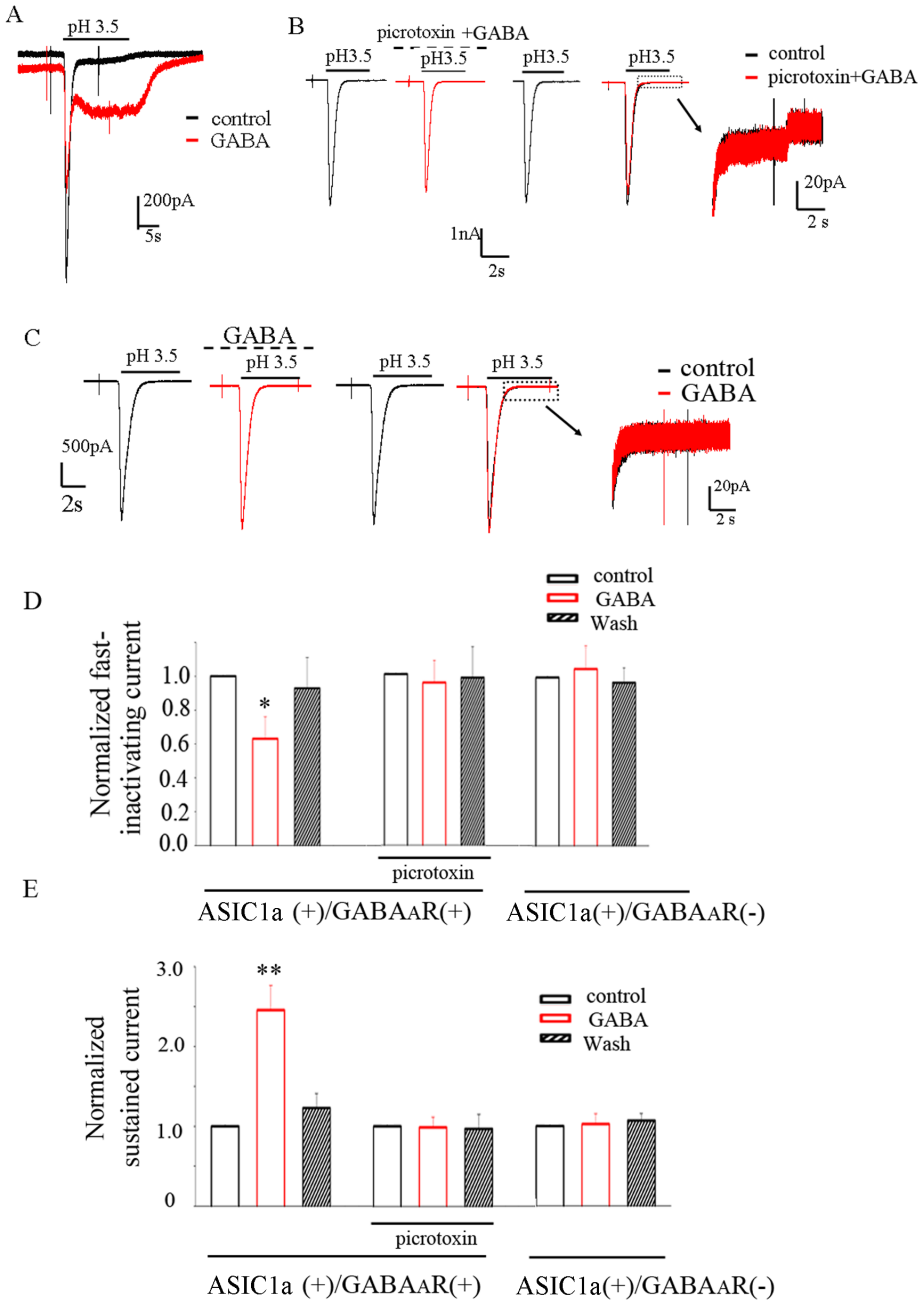
Activation of GABA_A_ receptors attenuated the peak current amplitude and enhanced the sustained current of ASIC1a. A, Example traces of a fast-inactivating transient current and a sustained current of ASIC1a activated by pH 3.5. GABA (100 μM) attenuated a fast-inactivating transient current and enhanced the sustained current of ASIC1a in HEK293 cells co-transfected with GABA_A_ receptor subunits (α_1_ and β_2_) and ASIC1a, which can totally abolished by co-application of picrotoxin (100 μM) with GABA. ASIC1a current traces were superimposed to the right (inset) (B). C, GABA had no effect on ASIC1a currents in HEK293 cells transfected with ASIC1a cDNA only. ASIC1a current traces were superimposed to the right (inset).

We used a whole-cell voltage-clamp configuration to record ASIC currents in HEK293 cells co-transfected with GABA_A_ receptor subunits (α_1_ and β_2_) and ASIC1a in response to repeated application of a pH 6 solution. The peak amplitude of whole-cell ASIC currents (evoked with pH 6 solution) in HEK293 cells was stable, averaging 2.51±0.37 nA (n = 38).Under our recording conditions the responses to GABA (at 100 µM) were small relative to ASICs currents (230±19 pA, n = 27) due to the small driving force on chloride at −60 mV(Figure 1A). Application of GABA reversibly inhibited ASIC currents (Figure 1A), which was largely abolished by application of a GABA_A_ receptors antagonist (either bicuculline or picrotoxin) (Figure 1B). To further confirm this phenomenon, we investigated whether GABA affected ASIC1a currents in HEK293 cells transfected with ASIC1a cDNA only. The result showed that GABA had no any effect on ASIC currents (Figure 1C). To clarify whether this inhibition is pH-dependent, we tested the effect of GABA on ASIC currents evoked by lowered pH (≤3.5). In general, the current evoked with pH 3.5 solution comprised of fast transient component and followed sustained component. Our results show that activation of GABA_A_ receptors also attenuated the peak current amplitude but enhanced the sustained current evoked with pH 3.5 solution, such effect was eliminated when GABA_A_R was blocked or HEK293 cells was transfected with ASIC1a cDNA only (Figure 2). These results suggested that activation of GABA_A_ receptors strongly regulates ASIC1a currents.

### Activation of ASIC1a modifies the current kinetics of GABA_A_ current

**Figure 3:**
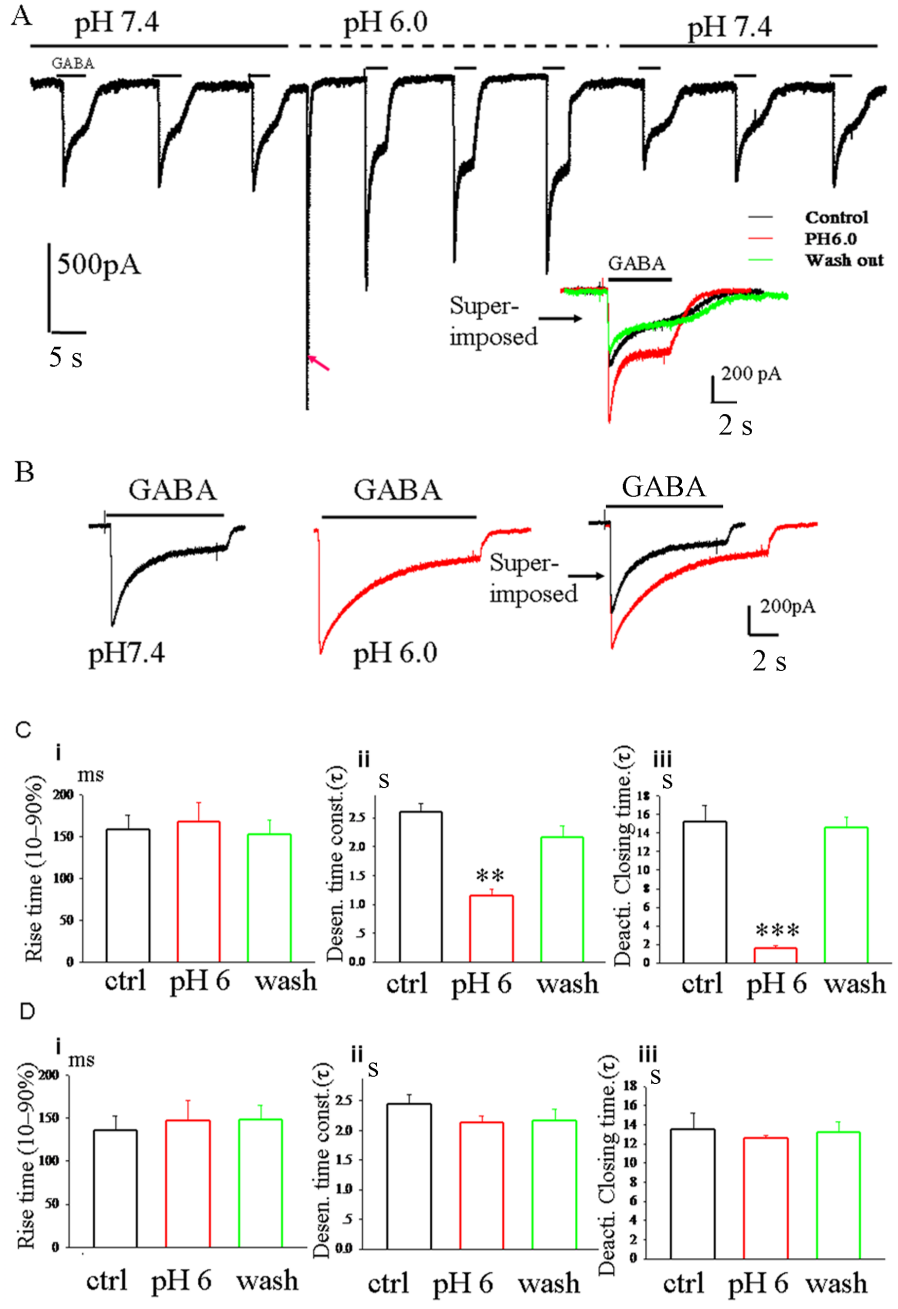
Activation of ASIC1a modifies the current kinetics of GABA_A_ current. A, The representative current traces recorded from HEK293 cells co-transfected with GABA_A_ receptor subunits (α_1_ and β_2_) and ASIC1a. ASIC1a activated by pH 6 reversibly altered the overall shape of GABA_A_ currents. GABA_A_ current traces were superimposed to the right. Red arrow indicates the current activated by pH 6 solution. B, pH 6 solution changed the peak current amplitude but not the shape of GABA_A_ currents in HEK293 cells transfected with cDNA of GABA_A_ receptor subunits only. C (cotransfected with both plasmids) and D (transfected with cDNA of GABA_A_ receptor subunits), bar graph showing the summarized data of rise time of activation (10–90%) (i), desensitization time constant (ii) and deactivation time (iii) of GABA_A_ currents in the presence of pH 7.4 or pH 6. ***, paired t-test, *p*<0.001, pH6 group vs. control group, n = 12.

Activation of ASIC1a reversibly altered the overall shape of GABA_A_ currents in HEK293 cells co-transfected with GABA_A_ receptor subunits (α_1_ and β_2_) and ASIC1a. Activation of ASIC1a had multiple effects on the GABA_A_ currents, not only was the peak amplitude of the ASIC current enhanced, but also the kinetics of the GABA_A_ currents were altered. Although activation of ASIC1a did not change the rise time (10%–90%) for the GABA_A_ currents, the time for desensitization or deactivation of GABA_A_ currents were markedly decreased when the pH of the extracelluar solution was decreased from 7.4 to 6. After washout, the time for desensitization and deactivation was totally recovered (Figure 3A, n = 12). To exclude the direct role of proton on GABA_A_ currents, we transfected HEK293 cells with GABA_A_ receptor subunits only and did not obtain any current response to pH 6 solution although the peak amplitude of GABA_A_ currents was also altered (Figure 3B, n = 12).These data indicate that the functions of GABA_A_ receptors are modified by ASIC1a.

### Co-immunoprecipitation of ASIC1a and GABA_A_ proteins in transfected HEK293 cells and primary cultured neurons

**Figure 4:**
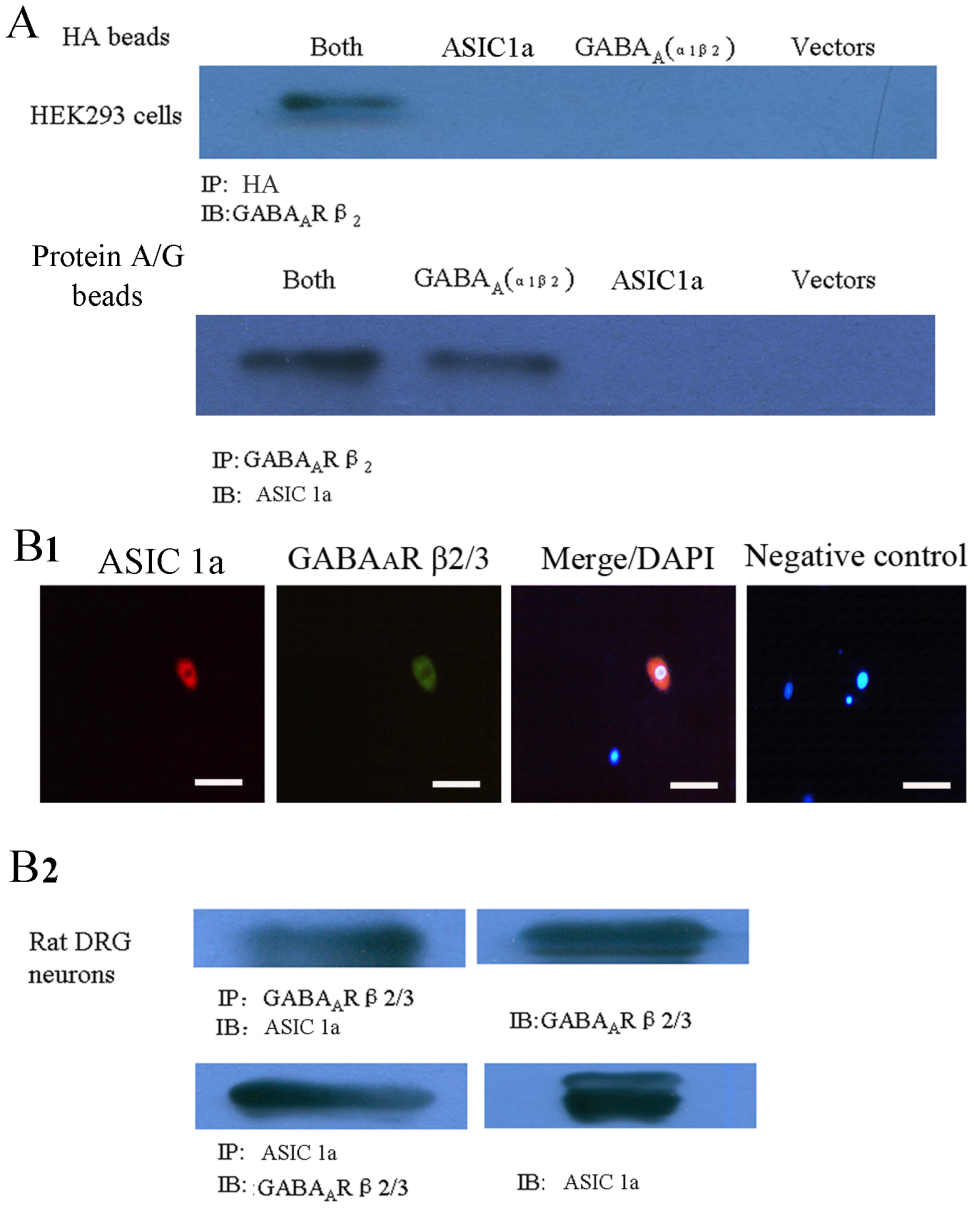
Co-immunoprecipitation of ASIC1a and GABA_A_ proteins in transfected HEK293 cells and DRG neurons. A. Co-immunoprecipitation of ASIC1a and GABA_A_ proteins in transfected HEK293 cells. GABA_A_ specifically co-precipitates with ASIC1a in cells co-transfected with ASIC1a and GABA_A_ (both). n = 3. B1. DRG neurons were double-labeled with anti-ASIC1a and -GABA_A_R antibodies. ASIC1a (red in left panel) as well as GABA_A_R (green in middle panel) localized to the apical membrane of DRG neurons. The merge (right) of ASIC1a and GABA_A_R images indicates that ASIC1a co-segregates with GABA_A_R in the apical membrane in neurons (yellow). Nuclei were identified by DAPI staining (blue). Scale bars equal 50 µm. B2. GABA_A_ precipitates with ASIC 1a in primary cultured DRG neurons. DRG neurons lysates were immunoprecipitated (IP) with anti-GABA_A_ β_2/3_ subunits polyclonal antibody in 8% SDS-PAGE gel, and the blot was then probed with anti- ASIC 1a polyclonal antibody (IB). In turn, ASIC 1a co-immunoprecipitates with GABA_A_. Cell lysates were immunoprecipitated with ASIC 1a antibody in 6% SDS-PAGE gel, and the blot was probed with GABA_A_ β_2/3_ antibody. These experiments were repeated three times with identical results.

To investigate the underlying mechanisms of interregulation of ASIC1a and GABA_A_ proteins, we transiently co-transfected ASIC1a and GABA_A_R in HEK293 cells. Due to endogenous expression of ASIC1a in HEK293 cells, we transfected ASIC1a with HA tag. In Co-IP experiments, anti-HA magnetic beads are used for the immunoprecipitation of specific HA-tagged proteins expressed in HEK293 cells. Our results showed that GABA_A_ specifically co-precipitated with ASIC1a only in cells co-transfected with ASIC1a and GABA_A_, which was confirmed by reversed Co-IP using antibodies to GABA_A_R β_2_ (Figure 4 A). ASIC1a endogenously expressed in HEK-293 cells [15]. Indeed, in our studies, we found that endogenous ASIC1a also co-precipitated with GABA_A_R in HEK293 cells transfect with GABA_A_R α_1_β_2_ subunits. It is well known that DRG neurons expressed both ASIC1a and GABA_A_R. To examine the possible co-expression of endogenous ASIC1a and GABA_A_R, primary rat DRG neurons were incubated with specific anti-GABA_A_R and anti-ASIC1a antibodies. The merged image indicates that ASIC1a and GABA_A_R are co-segregated with each other (Figure 4 B1). To further investigate a possible association between ASIC1a and GABA_A_R proteins, GABA_A_R was immunoprecipitated from rat DRG lysates with a polyclonal anti-GABA_A_R β_2/3_ antibody. The immunoprecipitated samples were probed with ASIC1a antibody. Conversely, the total DRG lysates was precipitated with ASIC1a antibody and then probed with GABA_A_R β_2/3_ antibody (Figure 4 B2). Taken together, our results showed that ASIC1a and GABA_A_ proteins co-immunoprecipitated each other.

### Interregulation of GABA_A_ receptors and ASIC1a in DRG neurons

**Figure 5:**
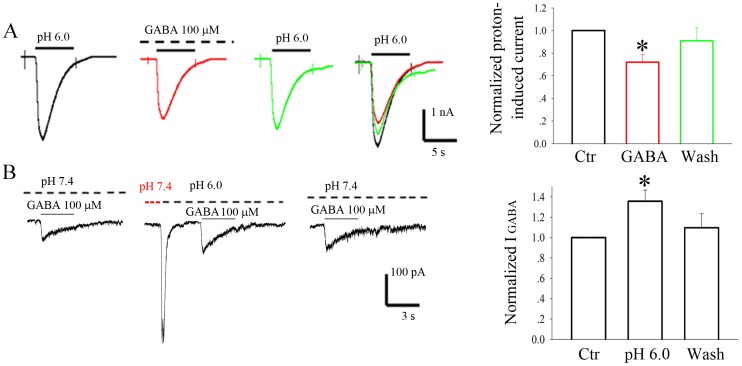
Interregulation of ASIC1a and GABA_A_ receptors in DRG neurons. A. Example traces of ASICs current activated by pH 6.0 solution in DRG neurons. GABA (100 μM) reversibly attenuated acid-evoked currents (n = 12). B. The representative current traces of GABA induced currents in DRG neurons. The pH 6.0 solution induced an inward current (enlarge inset). GABA-induced current was enhanced in the presence of pH 6.0 solution (n = 7).

Both GABA_A_ receptors and ASIC1a channels colocalized in rat DRG neurons. To examine the interaction of endogenous ASIC1a and GABA_A_ receptors in primary cultured rat DRG neurons, we used a whole-cell voltage-clamp configuration to record ASIC currents or GABA_A_ currents in DRG neurons in response to application of a pH 6 solution or 100 µM GABA. GABA (100 μM) reversibly attenuated acid-evoked currents (Figure 5A, n = 12). Conversely, GABA induced current was enhanced in the presence of pH 6.0 solution (Figure 5B, n = 7).

## Discussion

In the study reported here, activation of GABA_A_ receptors strongly attenuates the peak current amplitude of ASIC currents in transfected HEK 293 cells. Conversely, Activation of ASIC1a modifies the current kinetics of GABA_A_ current. These modifications included enhancement of the peak amplitude of GABA_A_ current and slowing of channel kinetics. Similar effects were observed in primary cultured DRG neurons. Furthermore, ASIC1a is co-segregated with GABA_A_ proteins in either transfected HEK 293 cells or rat DRG neurons, a finding verified by the immunoblotting assays. Our overall conclusion is that ASIC1a and GABA_A_ interregulated each other through a conformation-dependent protein-protein interaction.

### Interregulation of ASIC1a and GABA_A_ receptors through conformation-dependent protein-protein interaction

Modification of ASIC1a by GABA_A_ receptors occurred rapidly, and when the activation of GABA_A_ chloride channels were blocked by pharmacological blockade or genetic loss, the modifications were eliminated or largely reduced. The ASIC currents also recovered rapidly (Figure 1). This study suggests that ASIC1a current is modifed directly by activation of GABA_A_ receptors. In 2011, Cheng et. al. found that ASICs were reversibly inhibited by activation of GABA receptors in murine hippocampal neurons and such inhibition of ASICs required opening of the chloride channels. Therefore these authors speculate that a conformation-dependent interaction might occur between GABA receptors and ASICs [16]. Our present studies further demonstrated that these two receptors form a novel protein complex by performing the Co-IP experiments. These two receptors physically couple together and interact with each other that may depend on their conformation change. However, so far we can not exclude the possibility that an intermediate protein or regulator might participate in this receptor-receptor interaction.

Furthermore, in our present studies, we also demonstrated that pH 6 extracelluar solution largely decreased the time for desensitization or deactivation of GABA_A_ currents in HEK 293 cells co-transfected with ASIC1a and GABA_A_, but not in HEK293 cells transfected with GABA_A_ cDNA only, suggesting that these two receptors combine with each other and regulate each other. External protons regulate GABA_A_ receptor function by direct or allosteric interaction with the GABA binding site [14]. So far, we can not exclude the possibility that GABA_A_ receptor may have a proton binding site. In summary, upon binding of GABA, the GABA_A_ receptors undergo a conformational change and then modify current kinetics of ASIC1a by allosteric interaction with the proton binding site. Conversely, when ASIC1a is activated, the ASIC1a channels undergo a conformational change, then such change is converted to GABA_A_ receptors. Our results indicate that putative GABA_A_ receptors and ASIC1a channels phyically couple and functionally interact with each other, possibly via an inter-molecular association.

### Physiological and pathophysiological implications of the interaction between ASIC 1a and GABA_A_ receptors

of all ASICs, ASIC1a appears to play a prominent role in determining current amplitude and also affects the kinetics of H^+^-gated current [4], [17]–[19]. In CNS neurons, ASIC1a has been shown to be involved in synaptic plasticity, learning and memory [4], [20], and in acidosis-mediated, glutamate-independent neuronal injury [6], [21]. ASIC1a is expressed throughout the brain, with prominent expression in areas that receive rich synaptic input [17], [20], [22], [23]. Moreover, ASIC1a have a higher expression in GABAergic interneurons than that in the principal neurons [7], [24]. Given that the GABA_A_ receptors are the predominant inhibitory ionotropic receptors in the CNS, the interaction between ASIC1a and GABA_A_ receptors may occur at numerous locations and could be involved in a number of brain functions. Furthermore, ASIC1a is specifically located in DRG neurons and functions as a pain sensor, thus the interaction of GABA_A_ and ASIC1a may contribute to pain sensation.

## Materials and Methods

### Transfection of HEK293

HEK 293 cells were cultured in DMEM (HyClone) supplemented with 10% fetal bovine serum (HyClone), 1% penicillin/streptomycin at 37°C in a 5% CO_2_ incubator. Cells were transfected with pcDNA3.0 constructors encoding ASIC1a and/or GABA_A_R α_1_β_2_, using Lipofectamine TM 2000 (Invitrogen) according to the manufacturer's instructions. All recordings were made 24 to 48 h after transfection in the GFP-positive cells.

### DRG Cell Isolation and Culture

All the animal experiments were approved by the Medical Ethics Committee of Shandong University (number ECAESDUSM 2012029). Adult Wistar rats were euthanized by cervical dislocation and the entire spinal columns were removed. Bilateral DRGs were collected and washed twice with L-15 medium (Gibco, Gaithersburg, MD). They were then incubated in 10 ml L-15 medium containing 10 mg collagenase type 1 (Sigma, St. Louis, MO) and 0.25 ml 0.25% Trypsin (HyClone, Thermo scientific, USA) at 37°C for 50 min. DRGs were removed from the enzyme solution, centrifuged for 5 min at 1,000 revolutions/min, washed twice with L-15 medium, and transferred to 2 ml L-15 medium containing 10% FBS. The ganglia were triturated with a suction pipe for 3-min and then centrifuged for 50 seconds at 1,000 revolutions/min. Supernatants were placed into 35 mm diameter Petri dishes. The cells were then cultured at 37°C in a 5% CO2 incubator (Thermo Forma, Hamilton, NJ, USA). In our study, we chose freshly isolated neurons from rat DRGs in the range of 15–30 µm diameter to test the effect of GABA on acid-evoked currents or pH on GABA-induced current.

### Electrophysiological Recordings

Whole-cell voltage-clamp and current-clamp recordings were performed at room temperature (22–25°C) using a computer amplifier (Multiclamp 700B; Axon, New York, NY, USA) and a Digidata (1440A; Axon). Patch pipettes were filled with intracellular solution including (in mM): KCl 140, MgCl_2_ 2.5, HEPES 10, EGTA 11 and Na_2_ATP 5 with pH adjusted to 7.2 using KOH. Cells were bathed in extracellular saline containing (in mM): NaCl 150, KCl 5, CaCl_2_ 2.5, MgCl_2_ 2, HEPES 10, D-glucose 10 with pH adjusted to 7.4 using NaOH. The resistance of the recording pipettes was in the range of 5–8 MΩ. The series resistance was compensated for 70–80% after establishing a whole-cell configuration. The membrane potential was held at −60 mV throughout the recordings unless otherwise specified. Current-clamp recordings were obtained by switching to current-clamp mode after a stable whole-cell configuration was formed in the voltage-clamp mode. In this experiment, only cells with a stable resting membrane potential (less than −50 mV) were used. Signals were filtered at 4 kHz and then digitized at 10 kHz. The data were analyzed with the pCLAMP 10 acquisition software (Axon Instruments, CA, USA).

### Co-immunoprecipitation (Co-IP)

For Co-IP in human embryonic kidney 293 (HEK293) cells, Lipofectamine 2000 (Invitrogen) was used to transiently transfect ASIC1a with HA tag and/or GABA_A_R α_1_β_2_ following the manufacturer's instructions. Cell protein was purified for ASIC1a and GABA_A_R expression 36 h post-transfection. HEK293 cells were lysed in 1 ml of lysis buffer (1% Triton X-100, 50 mM Tris buffer pH 7.5) with a freshly added protein inhibitor mixture tablet. For Co-IP, the protein complexes were immunoprecipitated by anti-HA beads agarose (Sigma). After incubating, the beads were pelleted and washed three times in lysis buffer (1% Triton X-100, 50 mM Tris buffer pH 7.5), and the samples were loaded and run in 8% SDS-PAGE gel. The precipitates were transferred to a PVDF membrane and immunoblotted by anti-GABA_A_R β_2_ antibody (Millipore). The blots were developed by the enhanced chemiluminescence kit. Rat DRG or HEK293 cells were lysed in lysis buffer (1% Triton X-100, 50 mM Tris buffer pH 7.5) and a mixture of protease inhibitors. Anti-ASIC1a antibody or anti-GABA_A_R β_2/3_ antibody was immunoprecipitated with protein A/G-agarose beads (Santa Cruz Biotechnology). The protein complexes were immunoprecipitated with antibody cross-linked protein A/G-agarose beads. After incubating the lysates with cross-linked antibody, the beads were pelleted and washed three times in lysis buffer (1% Triton X-100, 50 mM Tris buffer pH 7.5), and the samples were loaded and run in 8% SDS-PAGE gel. The precipitates were transferred to a PVDF membrane and immunoblotted by anti-GABA_A_R β_2/3_ antibody (Millipore) or anti-ASIC1 antibody (Sigma). The blots were developed by the enhanced chemiluminescence kit.

### Drug Application

All drugs were purchased from Sigma-Aldrich Corp. (St. Louis, MO, USA). Except picrotoxin (dissolved in DMSO), all drugs were initially made up as stock solutions in distilled water and subsequently diluted in the external solution of the cells at a maximum of 1∶1000 to achieve their final working concentrations.

### Data Analysis

Data were expressed as mean ± SEM and compared statistically using paired *t* tests by Sigma Plot 10.0. A *p*<0.05 was required for the results to be considered statistically significant.
